# Setting the global research agenda for community-based HIV service delivery through the faith sector

**DOI:** 10.1186/s12961-021-00718-w

**Published:** 2021-05-17

**Authors:** Martha T. Ndlovu-Teijema, Maarten O. Kok, Sabine L. van Elsland, Hilleen Smeets, David Barstow, Lyn van Rooyen, A. M. van Furth

**Affiliations:** 1 Desmond and Leah Tutu Legacy Foundation, Cape Town, South Africa; 2 Department of Paediatric Infectious Diseases and Immunology, AI&II, Amsterdam University Medical Centre, Vrije Universiteit Amsterdam, Amsterdam, The Netherlands; 3 Erasmus School of Health Policy and Management at Erasmus University Rotterdam, Rotterdam, The Netherlands; 4 Department of Health Sciences, Vrije Universiteit Amsterdam, Amsterdam, The Netherlands; 5 Department of Paediatrics and Child Health, Tygerberg Hospital, Stellenbosch, University, Cape Town, South Africa; 6 MRC Centre for Global Infectious Disease Analysis, School of Public Health, Imperial College London, London, UK; 7 HIV and AIDS in 2030: A Choice Between Two Futures 2019, Corvallis, OR USA; 8 Van Rooyen info, Randburg, South Africa

**Keywords:** HIV, Health systems, Agenda-setting, Faith, Religion, Healthcare, Research priority-setting, Knowledge translation

## Abstract

**Background:**

While leading AIDS organizations expect faith and health collaborations to play a crucial role in organizing and scaling up community-based HIV services, it is unclear how this can be realized. Little primary research has been conducted into which strategies for collaboration and service provision are most effective, efficient, scalable and sustainable. Seeking to align research with urgent needs, enhance coordination and increase the likelihood that results are used, this study aimed to set an inclusive global research agenda that reflects priority research questions from key stakeholders at the intersection of HIV healthcare and faith.

**Methods:**

In order to develop this global research agenda, we drew from document analyses, focus group discussions, interviews with purposively selected key informants from all continents (policy-makers, healthcare providers, faith leaders, academics and HIV activists), an online questionnaire, and expert meetings at several global conferences. We carried out focus group discussions and interviews with faith leaders in South Africa. Other stakeholder focus groups and interviews were carried out online or in person in France, Switzerland, the Netherlands and South Africa, and virtual questionnaires were distributed to stakeholders worldwide. Respondents were purposively sampled.

**Results:**

We interviewed 53 participants, and 110 stakeholders responded to the online questionnaire. The participants worked in 54 countries, with the majority having research experience (84%), experience with policy processes (73%) and/or experience as a healthcare provider (60%) and identifying as religious (79%). From interviews (*N* = 53) and questionnaires (*N* = 110), we identified 10 research themes: addressing sexuality, stigma, supporting specific populations, counselling and disclosure, agenda-setting, mobilizing and organizing funding, evaluating faith-health collaborations, advantage of faith initiatives, gender roles, and education. Respondents emphasized the need for more primary research and prioritized two themes: improving the engagement of faith communities in addressing sexuality and tackling stigma.

**Conclusions:**

A wide range of respondents participated in developing the research agenda. To align research to the prioritized themes and ensure that results are used, it is essential to further engage key users, funders, researchers and other stakeholders, strengthen the capacity for locally embedded research and research uptake and contextualize priorities to diverse religious traditions, key populations and local circumstances.

## Background

Worldwide, 36.9 million people live with human immunodeficiency virus (HIV) [1, 2]. Services provided for people living with HIV include the provision of antiretroviral treatment, mental counselling, prevention, organization of adherence groups, home-based care and other medical and social support. Currently, 95% of these services are provided by medical staff and medical facilities [3]. In 2014, the United Nations (UN) launched fast-track goals to end AIDS, declaring that by 2030, 95% of the people living with HIV should know their status, 95% of those who know their status should be on antiretroviral treatment and 95% of those on treatment should be virally suppressed. According to the Joint United Nations Programme on HIV/AIDS (UNAIDS), community-based services for people living with HIV need to increase from approximately 5% to 30% by 2030 in order to achieve these goals [4].

One of the community structures expected to play an essential role in scaling up and sustaining community-based HIV services is the faith sector. Faith communities can be described as communities with a shared faith which can be characterized by beliefs in a higher power or order, a code or system that links values and actions and the idea that there is a reason and purpose to earthly existence [5]. Faith communities have a long-standing involvement in service provision for people living with and affected by HIV. These initiatives are very diverse in terms of scale, organization, populations reached and sustainability [6]. Initiatives include large-scale programmes that have been integrated into the health system for decades, short-term programmes financed through temporary funding from an international donor and local initiatives organized by a specific faith leader. The role of faith communities in providing HIV services is not undisputed. Regarding stigma and care for specific populations in particular, both positive and negative influences of the faith sector are seen [7]. Nevertheless, international organizations recognize the importance of involving the faith sector in providing services to people living with HIV and have developed and implemented a variety of approaches for collaboration [8–10].

While faith and health collaborations are expected to play a crucial role in organizing and scaling up community-based HIV services, it is unclear how this can be realized successfully. Faith initiatives are often embedded in local and social structures, making it difficult to generalize these individual initiatives to a broader context [11]. More research is needed to efficiently use available resources, support investments and make use of the community support that is needed for sustainable collaboration between the faith and healthcare sector [12, 13].

Little primary research has been conducted into which strategies for collaboration and service provision are most effective, efficient, scalable and sustainable. Current scientific publications about faith and healthcare collaborations consist mostly of overviews and observational studies [14]. Extensive grey literature on projects and programmes is available through organizations such as the Collaborative for HIV and AIDS, Religion and Theology (CHART). However, this literature does not provide insight into how current initiatives can be made more cost-effective, sustainable, integrated into systems and successful on a larger scale [15]. Moreover, different countries and populations have different research needs, and those which are most urgent for a specific setting are often unclear. Another unanswered question is how best to connect the various stakeholders working at the intersection of faith, healthcare and HIV. Research is thus needed that focuses on organizing primary research, connects different stakeholders and contributes to reproducing local successes on a larger scale and using the limited resources for research as efficiently as possible [16, 17]. As such, this study aims to set an inclusive global research agenda with a focus on cooperation between the faith and healthcare sector for organizing services for people living with HIV or at risk for HIV infection.

## Methods

**Fig. 1:**
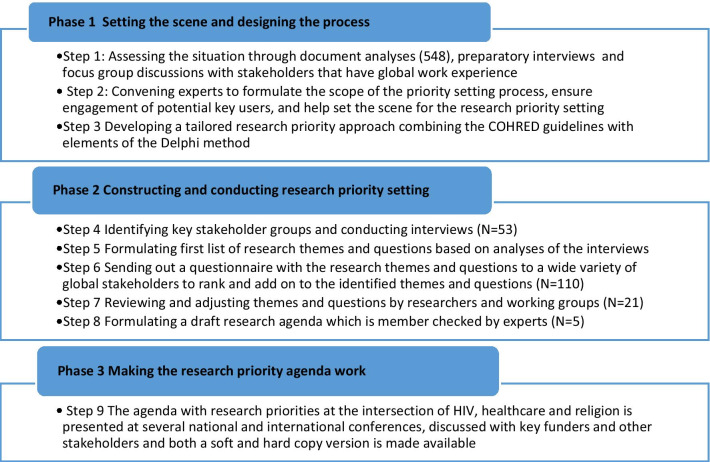
Phases and steps of the research priority-setting process

This research priority-setting process consisted of three phases and nine steps inspired by the priority-setting guidelines developed by the Council on Health Research for Development (COHRED) and other globally used priority-setting procedures (Fig. 1) [18, 19].

### Phase 1: Setting the scene and designing the process

#### Step 1

To assess the situation in which the priority-setting takes place and clarify the need for a priority-setting exercise at the intersection of healthcare and religion, we purposively sampled a core group of experts with extensive global work experience. These experts were approached by the researchers and asked to participate in an agenda-setting exercise. After initially approaching internationally recognized experts from the network of UNAIDS, World Council of Churches and known researchers in the field, through snowballing, more experts were approached. In addition, data was used from document analyses and five focus group discussions with faith leaders and healthcare workers (*N* = 170) in South Africa. We used the documents as input for designing the priority-setting process, to identify existing widely used concepts and to identify literature about the emerging themes (548 articles).

We interviewed selected experts through video calls or live from South Africa, the Netherlands, Switzerland and France, and used these preparatory interviews to establish a larger group of stakeholders, which was important for the priority-setting.

#### Step 2

To specify the scope, map and engage potential key informants and users and help set the scene for the priority-setting, we organized an expert meeting in Geneva with representatives from the World Council of Churches, UNAIDS, International AIDS Society (IAS) members and academia.

#### Step 3

Following the COHRED guidelines, we developed a tailored research priority-setting approach, for which we combined the Delphi [20] method with elements from the Child Health and Nutrition Research Initiative (CHNRI) method [21]. We defined our expected output as an inclusive global agenda, citing research priorities at the intersection of healthcare, HIV and religion. We aimed to bring together the expertise of scholars with in-depth knowledge of the existing research reservoir and the needs and experience-based knowledge of other stakeholders.

### Phase 2: Constructing and conducting research priority-setting

#### Step 4

In this next phase, we identified key stakeholders (policy-makers, healthcare providers, faith leaders, academics and HIV activists) from around the world, through purposive sampling and snowball sampling. Two researchers (MN, HS) interviewed the selected stakeholders about the need for research (*N* = 53). Interviews lasted between 45 and 90 min, took place either in person (*N* = 30) or via Skype (*N* = 23) and were audio-recorded and transcribed verbatim.

#### Step 5

A preliminary list of research questions and themes was identified by four researchers (MN, MK, SLvE, HS) after analysing and coding all interviews. Interviews were analysed using a constant comparative method of analysis and MAXQDA software [22].

#### Step 6

The list of research questions and themes was sent out by email as part of a digital questionnaire to purposively sampled key informants representing high-burden countries. Themes and questions were ranked, and potentially missing topics were identified (*N* = 110). The process did not yield any new themes.

#### Step 7

Based on the questionnaire outcomes, a specified list of questions and themes was compiled by three researchers (MN, MK, SLvE). These questions and themes were presented and discussed at two expert working groups at the IAS conference in Paris (*N* = 7) and a workshop organized by the University of Kwazulu-Natal, Collaborative for HIV and AIDS, Religion and Theology (CHART) (*N* = 14). Experts were from different high-burden countries and represented international organizations including the World Council of Churches, IAS, UNAIDS and several universities.

#### Step 8

Based upon the discussions in the expert working groups, a final research priority agenda was constructed by three researchers (MN, MK, SLvE). This agenda was member-checked and approved by five experts from the two working groups in step 7.

### Phase 3: Making the research priority agenda work

#### Step 9

As a final step, we closely collaborated with key funders, researchers, HIV activists and other key stakeholders to promote the use of the research priority-setting agenda. The agenda was presented at several national and international conferences, discussed with key stakeholders in planning meetings and in workshops and both a soft copy and printed booklet have been made available. In addition, collaborations with global partners such as the World Council of Churches, Christian AIDS Bureau for Southern Africa (CABSA) and others will ensure that the agenda is known and used in an international context.

Descriptive data from the questionnaires was analysed using the SPSS statistics package version 25.0 (IBM Corp., Armonk, NY, USA). This study was approved by the University of Cape Town Health Research Ethics Committee (Reference number: 123/2015). Written informed consent was obtained from all participants prior to the interviews, focus groups and questionnaires.

## Results

### Participants

**Fig. 2:**
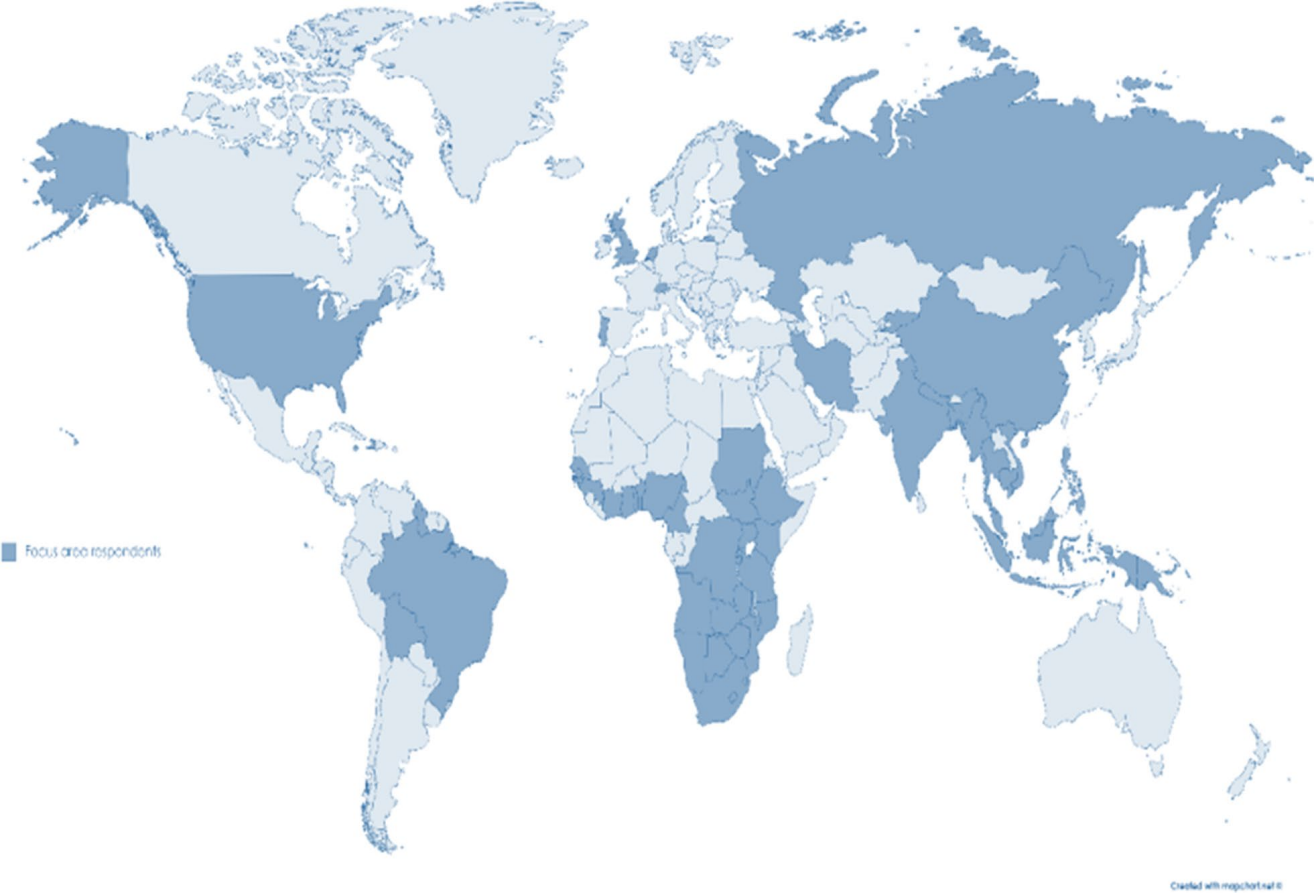
The 54 countries in which participants of the research priority-setting process worked

Initial research priorities and themes were identified in phase 2, step 4 (*N* = 53, data saturation after 47 expert interviews). The response rate to the questionnaire sent out in step 6 to rank the identified themes and priorities was 34.2% (*N* = 110 of *N* = 322 questionnaires sent out). The majority of the participants worked in Africa (70%) or on multiple continents (20%). Most participants worked in high-burden countries. The respondents worked in 27 of the 30 countries representing 89% of global HIV infection [23]. Figure 2 shows an overview of the countries in which respondents worked. The majority considered themselves religious (79%). Almost all respondents (92.1%) had experience working with specific populations (the top three of which were children and adolescents, women and girls, and sex workers).

Table 1 describes the key professional role(s) and work experience of participants. Respondents could report several professional roles at the same time. The primary professional role represents the role(s) with which participants currently identified. Many participants also had other relevant experience, such as experience as HIV activist, researcher, policy-maker/advisor, health service provider or as faith leader.

**Table 1:** Respondents’ roles (questionnaire and interview respondents) (respondents could choose multiple professional roles)

Respondents (*N* = 163)	Primary professional role(s) (%)	Experience with other relevant (professional) role (%)	Mean years of experience (min–max years)
Researcher (*N* = 107)	43	84	11 (1–41)
Policy-maker/advisor (* N* = 96)	28	72	9 (1–25)
Faith leader (*N* = 63)	35	35	18 (1–42)
Healthcare professional (*N* = 70)	23	60	15 (1–45)
HIV activist (*N* = 70)	36	50	12 (1–32)

### Key themes

Ten themes with specific research questions were identified and ranked and are presented in Table 2 according to ranking, starting with most prioritized themes.

**Table 2:** Research themes and questions presented in ranked order

Theme description	Theme questions
Theme 1: Addressing HIV-related stigma Stigma has been identified as a crucial aspect when researching faith and health collaborations. As such, working to understand and address stigma on different levels remains an important focus of any future research in this field. Stigma related to Christian faith communities has thus far been researched much more than stigma related to or expressed in other faith traditions. While there is existing research on stigma and its various forms, it is very clear from this current research priority-setting that there is still a great need for future research on the subject to assess the success of faith involvement in HIV services	-What is needed for faith communities to become a safe space within their community? -How does religion influence HIV-related stigma? -How can HIV-related stigma be reduced within faith communities? -How can HIV-related stigma be measured and quantified within faith communities? -How can faith communities speak about sexuality in a positive way? -How can faith communities help reduce stigma among families of people living with HIV?
Theme 2: Addressing sexuality within faith communities HIV and sexuality have always been important and charged topics when connecting healthcare and faith initiatives. The way in which faith institutions and leaders address sexuality and prevention of HIV infection can have a major impact, either positive or negative. As such, finding ways in which sexuality and prevention can be addressed in a positive light needs to be a primary focus for any future research on faith and health initiatives	-How do faith communities address sexuality? -How can faith leaders' attitudes towards sexuality and condom use be influenced to support the response to HIV? -How can religious organizations involve youth in addressing and promoting healthy sexual behaviour? -How can religious organizations address healthy sexual behaviour within their faith communities? -What kind of theological capacity needs to be developed for HIV education and prevention to be an integral part of the church, including education on sexuality? -What are the religious resources that would enable a religious community to talk about sexuality or sexual diversity? -How can education on healthy sexual behaviour be promoted from different faith traditions?
Theme 3: Researching how to organize the role of faith organizations in HIV initiatives The UN goal for scaling up community-based service delivery underscores the importance for community structures such as faith structures in identifying possible collaborations with the healthcare sector and entry points for these collaborations. While the research available states the importance of faith interventions, comparisons between interventions are rarely available. To further advance the field, research should focus on the steps that come after identifying the importance of faith involvement	-What is needed for diverse religious organizations to collaborate on HIV-related initiatives? -What do diverse religious organizations need to support HIV treatment and adherence? -What do diverse religious organizations need to support HIV surveillance? -What do diverse religious organizations need to be involved in HIV-related palliative care? -What is needed to include religious structures (faith-based organizations [FBOs], faith leaders, health services) in improving HIV-related initiatives? -What elements of faith traditions can contribute to health initiatives? -What in the faith tradition conflicts with health initiatives?
Theme 4: Addressing gender roles concerning HIV initiatives HIV-related healthcare and gender roles are inevitably tied. More in-depth research needs to be conducted on the role that faith and faith initiatives can play in addressing HIV risk and prevention as it pertains to ideas on gender and behaviour	-How can faith communities influence young people’s ideas on gender roles? -How can faith/health initiatives influence ideas about gender roles to improve HIV-related healthcare? -How can faith leaders help include men in providing HIV-related healthcare? -How can faith communities encourage men and boys to seek HIV-related healthcare?
Theme 5: Educating faith communities about HIV The education of faith communities about HIV is a top priority for any faith and health collaboration. This education, in line with UN fast-track goals, should focus on different aspects and include behavioural, medical and structural prevention. Prevention as part of education is seen as an important but often practically challenging part of faith involvement. In addition to educating faith communities about HIV, it should be explored whether there is a corresponding need to educate healthcare workers or institutes about the possibilities of the faith sector providing community-based HIV service	-How can education for key actors involved in faith/health collaborations be efficiently organized and financed? -What is the best way to educate faith leaders on how health systems function? -What is the best way to educate health professionals about the role of religion in HIV-related healthcare and to connect with faith leaders? -How can education on how antiretrovirals work be integrated into current education for faith leaders? -How can faith communities be educated about HIV training and information when faith leaders want to support people living with HIV? -How can education on disclosure be included in the training of faith leaders? -In what ways can faith leaders be supported and motivating with respect to educating communities about HIV?
Theme 6: Keeping HIV on the global agenda through community support Community organizations, including religious and faith organizations, are important actors in the field of HIV. How can community support with a focus on faith initiatives play a role in keeping HIV as an important agenda item for policy-makers, international organizations and other stakeholders? While advocacy organizations with a faith background have been working on agenda-setting, little research has been conducted on the success of this work	-What are ways in which national and international (healthcare) organizations can connect with faith leaders? -Which issues can national and international (healthcare) organizations use as a means of connecting with faith leaders? -What is the current position of national AIDS councils? -What are successful examples of advocacy and how can these be scaled up? -What is the role of faith-based organizations in HIV-related policy advocacy? -How can HIV-related health services be integrated into the agenda of religious organizations? -How can faith leaders be supported in forging networks to influence agenda-setting on HIV?
Theme 7: Strategies for mobilizing and organizing funding in a changing funding environment HIV funding has been globally reprioritized, and there has been a shift towards less vertical and more integrated HIV funding programmes. At the same time, ministries of health are not used to fund activities that are organized by the faith sector, such as community-based HIV service delivery. Research is needed on how faith and faith initiatives can play a role in mobilizing HIV funding and how funding for faith and health collaboration can best be organized	-How can service provision of HIV services be guaranteed by funding FBOs? -How can service provision of HIV services be guaranteed through government funding? -How can service provision of HIV services be guaranteed by national and international private funders? -What is the effect of different incentives on voluntary caregivers from FBOs? -What can be done to coordinate funding efforts to be more effective?
Theme 8: Creating an environment for counselling and disclosure with faith leaders Counselling and disclosure are important with respect to improving medication adherence and acceptance for people living with HIV. Faith leaders can play an important role in this, either positively or negatively. While stakeholders recognize the role that faith leaders have in creating a safe space for counselling and disclosure, more research is needed to determine how this can be achieved in a sustainable and inclusive way	-What approach is best for connecting with faith leaders who do not believe in HIV education? -What are possible roles for faith leaders or pastoral workers in counselling people living with HIV? -How can faith leaders counsel and support young people living with HIV? -How can faith leaders/religious organizations support disclosure of HIV status? -How can faith leaders and pastoral workers be supported in providing HIV-related counselling? -How can faith leaders prevent people living with HIV from dropping out of church/mosque/synagogue/temple after they disclose their status to the community?
Theme 9: Monitoring and evaluating faith/health initiatives Faith and health initiatives are present in different forms and on different levels. While some programmes are conducted thorough monitoring and evaluation, faith and health collaborations are often not monitored or evaluated in ways that are familiar to funders and researchers. Monitoring and evaluation are key to understanding impact, improving existing programmes and identifying sustainable collaborations for the future	-What kind of monitoring and evaluation is needed to evaluate the impact of the faith response to HIV? -How can the documentation and data collection of current faith/health initiatives be optimized on different levels? -How can promising faith/health initiatives be adapted to local settings or scale? -How can local initiatives to improve sustainability be recognized and supported? -What role does media play in HIV-related faith/health initiatives? -Which faith communities are most likely to benefit from HIV training programmes?
Theme 10: Supporting specific populations through faith initiatives Specific or key populations are an important focus of many HIV-related programmes. This theme aims to research what role faith and faith initiatives can play in this regard. It is evident that faith and specific key populations, such as men who have sex with men, or sex workers, have an ambiguous relationship. Faith organizations have been reported to have both a positive and negative impact on key HIV populations. The question of whether specific populations should be a focus of faith and health collaborations or—as stated by current UN goals—the focus of an inclusive approach that also addresses specific populations remains unanswered	-How can the faith sector support people living with HIV in prisons? -How can religious organizations support children affected by the HIV epidemic (including noninfected orphans)? -To what extent do faith/health initiatives need to be adapted to reach specific key populations? -How can the faith sector support women and girls affected by the HIV epidemic? -How can faith communities be educated on the needs of HIV-positive children/youth? -How can children who are born HIV-positive be reached and supported in a church setting?

### Priority themes: addressing sexuality and stigma

Participants consistently prioritized two research themes: addressing sexuality and addressing stigma. Participants acknowledged that while a lot of research has focused on stigma and sexuality, these themes remain a clear priority for the future. Research questions focusing on how specific elements of faith traditions enable or hamper health service delivery (including addressing sexuality and key populations) were identified as important and specifically highlighted during expert meetings. Researcher: “If you can find theological ways of speaking about sexuality, you can speak about sexuality in the church. So, religion is a doorway. It is a gatekeeper, but it is also a doorway.”

### Should prevention be a separate theme?

During several interviews and expert meetings, participants discussed the role of the faith sector in prevention and the need for research on prevention. Some argued that prevention should be a separate theme, whereas others considered prevention as part of the remaining themes identified. Participants linked this discussion to the debate about the role of the faith sector in prevention. Several faith leaders and policy-makers did not consider prevention to be something that should be addressed by faith and health collaborations. Policy-maker:

What they the church did was, they thought “What can we do?”, instead of “What can’t we do?” What they can do is they can support the inclusion of people living with HIV. They can provide religious support [….] They can support and promote treatment, so they can do that. […..] they can promote testing. What they cannot do is prevention. So they ignored it. They basically said, “Okay, we are not touching that. We are not going to say it is good or bad […..] we are going to the areas that we can go”. And that has been very interesting.

### Improving the use of research

Many participants pointed out that there is not just a need for more primary research, but also a need for better dissemination and use of available research and more cooperation between different stakeholder groups. Participants pointed out that while some themes have been studied for years, many findings remain unused because they do not reach those who could benefit from them. Participants argued that future efforts should therefore not just focus on conducting more and better research, but also on strengthening local research capacities, engaging key stakeholders in research formulation, interpretation and use, strengthening infrastructure for sharing results and best practices, and collaboration between international and local stakeholders.

## Discussion

The research agenda provides an overview of the research priority themes and questions for faith involvement in service delivery for people living with HIV. It is a combined agenda representing the views of different stakeholders from various parts of the world. While the themes and questions presented reflect a wide variety of responses, some of the themes and questions will fit certain regions better than others.

Participants consistently prioritized tackling stigma and addressing sexuality. While useful research has been conducted into these themes, new locally led and locally specific research and improved dissemination of results is clearly needed to better address sexuality and stigma-related research questions [24, 25]. Also, research shows that a constant focus on existing policy and programs is needed in order to not lose achieved progress [26]. Faith initiatives concerning HIV and messaging about sexuality remain controversial and under-researched [27]. Specific populations and faith initiatives have a very ambiguous history, with some initiatives increasing stigma and some diminishing it [28, 29]. In addition, this study shows that experts questioned whether a tailored approach to diminish stigma for specific populations is needed or that an all-inclusive approach will help diminish overall stigma, including for specific populations. There is a debate as to whether faith leaders themselves can or should play a role in addressing sexuality, or whether they should collaborate with others who can address these issues more effectively. Research should focus on clarifying and evaluating these roles and the possibilities for faith communities in addressing stigma and sexuality.

Several themes of the research agenda focus on issues that are considered part of health systems research, such as mobilizing and allocating funding for HIV collaboration and organizing the role of faith organizations in HIV initiatives [30]. Shifting HIV services from healthcare to community structures requires a shift in funding for these activities. In addition, funding for HIV programmes is shifting from vertical to integrated funding for health systems [25]. More health systems research is needed to explore which strategies for mobilizing and allocating funding work best. The new financing possibilities also raise the important question of which structure has to take ownership for specific parts of HIV service provision—a question that needs to be answered to make future programmes successful and sustainable.

HIV prevention was not identified as a separate theme in this research priority-setting, despite being described in literature as an important focus for future faith involvement [31]. Some respondents consider faith involvement in HIV prevention as controversial and feel that it should therefore not be a current focus of research. Even in countries such as Brazil, where faith-based organizations are highly integrated into service delivery for people living with HIV, prevention services are generally not carried out by these organizations. Faith initiatives focusing on HIV prevention often encounter stigma, and therefore, prevention remains a difficult topic to address in faith and healthcare collaborations. Literature shows that faith programmes addressing prevention usually focus more on abstinence and less on combination prevention [27]. In order to achieve the fast-track goals, however, it is vital that prevention services are scaled up, and the faith sector has huge potential for contributing in this regard [32, 33].

An important outcome of this agenda is the need for more stakeholder collaboration and improved governance and uptake of research. The faith sector, healthcare sector and academic world have their own goals and systems of communicating and disseminating knowledge. The diverse goals and systems make it a challenge to connect research with needs from the field and support the use of results [34, 35]. Faith structures that have programs focusing on HIV and AIDS rarely research the effectiveness and lessons learned; collaboration with other stakeholders would make this possible. In addition, comparative studies of different faith programmes addressing HIV are very rare, and there is little collaboration and evaluation, whereas this is needed to scale up and reach international goals [36].

There is a clear need for more demand-driven and locally led research in high-burden countries, and further development of a communication infrastructure for sharing best practices and lessons learned [25]. To develop and support such demand-driven and locally led research and increase the likelihood that results are used, a systemic approach is required [37–39].

While foreign donors can support such research, it is also essential to build a local “sponsorship constellation” that mobilizes local funding for research and legitimates the role of research in society [39, 40], while also monitoring to what extent results are used, and ensuring that local practices are considered for those who interpret findings and offer technical guidance [41, 42].

Available research on faith and health initiatives has mostly been conducted in Christian, English-speaking countries in sub-Saharan Africa [13, 43]. While this agenda emerged from a wide variety of interviews and questionnaires, the research priorities might tend towards needs that are specific to this region and its corresponding traditions. With 19.6 million of the 36.9 million people worldwide living with HIV in 2017 (53.1%), Eastern and Southern Africa represent an important focus area for future HIV research [1].

## Conclusions

The research priority agenda presented here aims to provide an overview of the research most needed at the intersection of healthcare and religion globally. The great diversity in both religious traditions and healthcare involvement should be considered when interpreting this agenda. In addition, given that the HIV epidemic impacts different populations in different countries and regions, it is vital that the priorities are contextualized.

While some priority areas might concern research topics for which there is existing research, our data showed that there is an urgent need for new primary research that focuses on core questions from the field. This agenda allows researchers and their funders to align research with current needs. People living with HIV and their representatives, policy-makers and civil society organizations can help attune research to these priorities, inform actual studies and support the translation of results into action.

## Data Availability

The datasets used and/or analysed during the current study are available from the corresponding author on reasonable request. Due to the specific terms of the ethical clearance for this study, data is not publicly available.

## References

[1] UNAIDS. Joint United Nations Programme on HIV/AIDS. Fact sheet world AIDS day 2018 [document on the internet]. Geneva Switzerland; 2018. http://www.unaids.org/sites/default/files/media_asset/UNAIDS_FactSheet_en.pdf. Accessed 10 Feb 2020.

[2] World Health Organization (WHO). HIV/AIDS data and statistics [document on the internet] 2018. https://www.who.int/hiv/data/en/. Accessed 10 Jan 2020.

[3] UNAIDS. Joint United Nations Programme on HIV/AIDS. 90–90–90, an ambitious treatment target to help end the AIDS epidemic [document on the internet]. Geneva Switzerland; 2014. http://www.unaids.org/en/resources/documents/2014/90-90-90. Accessed 10 Feb 2020.

[4] UNAIDS. Joint United Nations Programme on HIV/AIDS. Fast track ending AIDS epidemic by 2030 [document on the internet]. Geneva Switzerland; 2014. http://www.unaids.org/sites/default/files/media_asset/JC2686_WAD2014report_en.pdf. Accessed 10 Feb 2020.

[5] Loue S. Faith Community. In S. Loue (Ed.), Mental Health Practitioner’s Guide to HIV/AIDS: (pp. 215–216). New York: Springer. 2013.

[6] Wodon Q, Olivier J, Tsimpo C, Nguyen M (2014). Market share of faith inspired healthcare providers in Africa. Rev Faith Int Affairs.

[7] Schmid B, Thomas E, Olivier J, Cochrane JR. The contribution of religious entities to health in sub-Saharan Africa. 2008 study commissioned by B & M Gates Foundation.

[8] GFTAM. The Global Fund to fight AIDS, Tuberculosis and Malaria. Report on the involvement of faith-based organisations in the Global Fund [document on the internet]. Geneva Switzerland; 2008. https://s3.amazonaws.com/berkley-center/GlobalFundReportInvolvementFaith-BasedOrganisations.pdf. Accessed 10 Feb 2020.

[9] UNAIDS. Joint United Nations Programme on HIV/AIDS. Partnership with faith-based organisations: UNAIDS strategic framework [document on the internet]*.* Geneva Switzerland; 2009. http://data.unaids.org/pub/report/2010/jc1786_fbo_en.pdf Accessed 10 Feb 2020.

[10] UNFPA. The United Nations Populations Fund Guidelines for engaging faith-based organisations (FBO’s) as agents of change [document on the internet]. New York; 2009. https://www.unfpa.org/sites/default/files/resource-pdf/fbo_engagement.pdf Accessed 10 Feb 2020.

[11] Olivier J, Wodon Q. The role of faith-inspired health care providers in Sub-Saharan Africa and Public-Private partnerships. Strengthening the Evidence for Faith-inspired Health Engagement in Africa*,* Volume 2. 2012. Washington DC: The World Bank, HNP discussion papers.

[12] Olivier J, Wodon Q. The role of faith-inspired health care providers in Sub-saharan Africa and Public-Private partnerships. Strengthening the Evidence for Faith-inspired Health Engagement in Africa*,* Volume 3. 2012. Washington DC: The World Bank, HNP discussion papers.

[13] ARHAP-WHO African Religious Health Assets Programme. Appreciating Assets: The Contribution of Religion to Universal Access in Africa [document on the internet]. Cape Town South Africa; 2006. Accessed 10 Feb 2020.

[14] Olivier J, Wodon Q. The role of faith-inspired health care providers in Sub-Saharan Africa and Public-Private partnerships. Strengthening the Evidence for Faith-inspired Health Engagement in Africa, Volume 1. 2012. Washington DC: The World Bank, HNP discussion papers

[15] Olivier J, Haddad B, Leonard G, Schmid B. *The cartography of HIV and AIDS, religion and theology: a partially annotated bibliography [document on the internet].* The Collaborative for HIV and AIDS, religion and theology (CHART). Pietermaritzburg South Africa 2016. http://chart.ukzn.ac.za/images/downloads/CHART_XII_bibliography.pdf Accessed 10 Feb 2020.

[16] Olivier J, Smith S (2016). Innovative faith-community responses to HIV and AIDS: summative lessons from over two decades of work. Rev Faith Int Aff.

[17] Francis S, Liverpool J (2009). A review of faith-based HIV prevention programmes. J Relig health.

[18] Montorzi G, de Haan S, Ijsselmuiden C. Priority setting for research for health, a management process for countries [document on the internet]. COHRED council on health research for development 2010. http://www.cohred.org/downloads/Priority_Setting_COHRED_approach_August_2010.pdf. Accessed 10 Feb 2020.

[19] Viergever R, Olifson S, Ghaffar A, Terry R (2010). A checklist for health research priority setting: nine common themes of good practice. Health Res Policy Syst.

[20] Yoshida S (2016). Approaches, tools and methods used for setting priorities in health research in the 21^st^ century. J Global Health..

[21] Rudan I, El Arifeen S, Black R. A systematic methodology for setting priorities in child health research investments*.* Child Health and Nutrition Research Initiative (CHNRI): A new approach for systematic priority setting. 2006 Dhaka Bangladesh.

[22] Pope C, Ziebland S, Mays N (2000). Qualitative research in health care: Analysing qualitative data. Br Med J.

[23] UNAIDS. Joint United Nations Programme on HIV/AIDS. Understanding fast-track, accelerating action to end the AIDS epidemic by 2030 [document on the internet]. Geneva Switzerland; 2015. http://www.unaids.org/sites/default/files/media_asset/201506_JC2743_Understanding_FastTrack_en.pdf Accessed 10 Feb 2020.

[24] Tomkins A, Duff J, Fitzgibbon A, Karam A, Mills E, Munnings K (2015). Faith-based health care 2: Controversies in faith and healthcare. Lancet.

[25] WHO World Health Organisation Global Health Sector Strategy on HIV 2016–2021; towards ending AIDS [document on the internet] Geneva Switzerland; 2016 [Cited 15^th^ March 2021] https://apps.who.int/iris/bitstream/handle/10665/246178/WHO-HIV-2016.05-eng.pdf;jsessionid=BC6FD2EEE4556B7174E4EABB087F48E4?sequence=1

[26] Cueto M, Lopes G (2021). Backlash in global health and the end of AIDS’ exceptionalism in Brazil, 2007–2019. Glob Public Health..

[27] Cornelius JB, Appiah JA (2016). Literature: a 5 year review of faith based sexuality education and HIV prevention programs. Curr Sex Health Rep.

[28] Garcia J, Parker R (2011). Resource mobilization for health advocacy: Afro-Brazilian religious organisations and HIV prevention and control. Soc Sci Med.

[29] Campbell C, Skovdal M, Gibbs A (2011). Creating social spaces to tackle AIDS-related stigma: reviewing the role of church groups in sub-Saharan Africa. AIDS behav..

[30] Powell TW, Weeks F, Illangasekare S, Rice E, Wilson ED, Hickman D, Div M, Blum R (2017). Facilitators and barriers to implementing church-based adolescent sexual health programs in Baltimore city. J Adolesc Health.

[31] Ochillo MA, van Teijlingen E, Hind M (2017). influence of faith-based organisations on HIV prevention strategies in Africa: a systematic review. Afr Health Sci.

[32] UNAIDS. Joint United Nations Programme on HIV/AIDS. Combination prevention: Tailoring and coordinating Biomedical, Behavioural and structural strategies to reduce new HIV infections [document on the internet]. Geneva Switzerland; 2010. http://files.unaids.org/en/media/unaids/contentassets/documents/unaidspublication/2010/JC2007_Combination_Prevention_paper_en.pdf. Accessed 10 Feb 2020.

[33] Duff J, Buckingham W (2015). Faith-based health care 3 Strengthening of partnerships between the public sector and faith-based groups. Lancet.

[34] Caplan N (1979). The two-communities. Theory and knowledge utilization. Am Behav Sci.

[35] Pisani E, Kok M (2016). In the eye of the beholder: to make global health estimates useful, make them more socially robust. Glob Health Action.

[36] Wingood GM, Robinson LR, Braxton ND, Er DL, Conner AC, Renfro TL, Rubtsova AA, Hardin JW, DiCelemente RJ (2013). Comparative effectiveness of a faith-based HIV intervention for African American women: importance of enhancing religious social capital. Am J Public Health.

[37] Pang T, Sadana R, Hanney S, Bhutta Z, Hyder A, Simon J (2003). Knowledge for better health: a conceptual framework and foundation for health research systems. Bull World Health Organ.

[38] Kok MO, Gyapong JO, Wolffers I, Ofori-Adjei D, Ruitenberg J (2016). Which health research gets used and why? An empirical analysis of 30 cases. Health Res Policy Syst.

[39] Kok MO, Gyapong JO, Wolffers I, Ofori-Adjei D, Ruitenberg EJ (2017). Towards fair and effective North-South collaboration: realising a programme for demand-driven and locally led research. Health Res Pol Syst.

[40] Kok MO, de Souza DK (2010). Young voices demand health research goals. Lancet.

[41] Hegger I, Kok MO, Janssen SWJ, Schuit AJ, van Oers HAM (2016). Contributions of knowledge products to health policy: a case study on the Public Health Status and Forecasts Report 2010. Eur J Public Health..

[42] Kok MO, Bal R, Roelefs CD, Schuit AJ (2017). Improving health promotion through central rating of interventions: the need for Responsive Guidance. Health Res Pol Syst.

[43] Olivier J, Tsimpo C, Gemignani R, Shojo M, Coulombe H, Dimmock F (2015). Faith-based healthcare. 1 Understanding the roles of faith-based health-care providers in Africa: review of the evidence with a focus on magnitude, reach, cost, and satisfaction. Lancet..

